# National Dental Association

**Published:** 1898-12

**Authors:** 


### Reports of Society Meetings.

### NATIONAL DENTAL ASSOCIATION.

(Continued from page 732.)

### DISCUSSION.

Discussion of Dr. B. Holly Smith's paper on "Removal of the Dental Pulp."

(For abstract of paper, see page 702.)

*Dr. Barrett.*—There is much in the subject which needs clearing up. The author of the paper attributes the death of the pulp under arsenical application to the action of the arsenic on nerve tissue. Arsenic is a corrosive agent; it induces necrosed conditions of osseous tissue. If death is due to constriction at the apical foramen from forced stasis, how is it caused when there is no open foramen? If the arsenic is not securely sealed in the tooth cavity, the effects are just as fatal upon the buccal tissues as upon the pulp itself.

*Dr. Hungerford.*—The spirit of the paper was not an exemplification of pathological conditions, the use of therapeutic agents and surgery being the key-note. I am opposed to the contamination of the blood-stream by the introduction of therapeutic agents, as by hypodermic injections. I treat pulps surgically, varying the method according to conditions. After the application of arsenic to the pulp, death is due to congestion in the pulp-chamber, not at the apical foramen.

*Dr. Patterson.*—Pericemental inflammation after pulp removal can always be avoided by removing the pulp by surgical methods. Cocaine can be forced into the pulp until it is completely anæsthetized, using very finely pulverized crystals of cocaine. With the chloride of ethyl spray the pulp can be so congealed that cocaine can be forced into it by means of pressure with punk, cotton, or gutta-percha. The pulp can then be removed absolutely without pain.

*Dr. Taft.*—I abandoned the use of arsenic twenty years ago. Its effects are objectionable not only upon the pulp itself, but extending to other tissues. The results are too uncertain. With increase of facilities other methods are so efficient that there is no longer any excuse for its use. Cocaine, finely pulverized, is readily taken into the pulp by absorption until it is so anæsthetized that it can be removed with as little pain as a paring from the finger-nail. When the pulp is removed by surgical procedure and all soft tissues removed from the canal, and hemorrhage arrested, then is the time to close it up. Seal it up immediately, and there is no liability of any change pernicious to its welfare.

*Dr. Watkins.*—When I have a large cavity with free access, I prefer to punch the pulp out with a slim piece of orange-wood. It is instantaneous; the pulp is removed entire, and the tooth is ready for filling.

*Dr. Barrett.*—Those who oppose arsenic as a dangerous drug advise the use of cocaine! Which is the most dangerous? It is "jumping from the frying-pan into the fire."

*Dr. B. H. Smith.*—The surgeon who uses cocaine knows the liabilities and is prepared to combat evil effects. The trouble with arsenic is that we do not know what the results may be.

Dr. Crawford spoke of the importance of preventing septic infection (which he prefers to call *toxic invasion*) by stopping each root as soon as the canal is emptied, not leaving it open to serve as a means of self-destruction while operating in the other canals.

Dr. Smith, in closing the discussion, said that he had desired to present the record of some clinical cases, but had concluded to wait and study them further.

REPORTS OF SECTIONS.

Section I. recommends that chemistry be attached to Section V., as the little that has been offered during the past year has been so interwoven with therapeutics that it properly belongs to Section V.

Empiricism holds a powerful sway in prosthetic dentistry, and until more attention is given to the science of prosthesis and less to the detail of mechanical procedure it cannot hope to occupy a prominent place in the national body.

Section I. presented a paper by Dr. L. E. Custer, entitled "Recent Improvements in the Electric Oven, and Methods of determining the Heat in the Fusion of Porcelain," of which the following is an abstract:

In the improved oven the perfection of detail has all been in the direction of producing the highest heat in the oven cavity, with the lowest possible heat of the platinum wire, which in the Custer oven has been reduced step by step by perfecting every available point that might be of advantage, until it is so reduced that it will now stand hundreds and perhaps thousands of heatings.

Dr. Thomas reports having used nearly one hundred ounces of Close material without a burn-out, a conservative estimate of which would be that the oven was heated from eight hundred to one thousand times. This result has been attained by making every inch of the walls heat-producing surfaces (except the two small openings), arranging wires over the entire surface as closely together as can be without touching laterally, the wires being also brought to the surface of the clay, placing them only deep enough to be caught, so that nothing intervenes between the wire and the object heated. The wires are also placed closer together as the distance from the centre increases, so that all portions of the cavity are equally heated. This idea, original with Dr. Custer, is now being copied in electric culinary utensils. The discovery of the fact that the negative end of the wire becomes about one-fifth hotter in a constant electric current than the positive, led to the method of winding the oven with the negative end of the wire more extensively than the positive, and solves the most perplexing problem, and the oven may now be said to be perfect in every detail.

Dr. Custer described two methods for determining the heat of the oven. One is by the use of a thermometer, which he inserts in a clay cup outside of the oven, having a twenty-gauge platinum wire, of which one end enters the oven cavity, the other terminating in the cup which encloses the bulb of the thermometer, the heat of the cup end being always exactly proportionate to the end of the wire in the oven.

Another and better method—one under complete control—is by the use of an arc light. By means of a little frame, placed on the top of the oven, a small carbon is held opposite the upper opening, another carbon, which is movable, being held in a guide. When it is desired to see the condition of the porcelain the movable carbon is pushed towards the fixed carbon until the two are in contact.

The movable carbon is then withdrawn a short distance, and a brilliant arc will be struck, throwing such a strong light in the oven that the operator sees the porcelain melt as distinctly as if in the open air. There is no guesswork, and the eye is relieved of the great strain of trying to see an indistinct object.

DISCUSSION.

In the discussion of Dr. Custer's paper, Dr. H. A. Smith asked if a method had been discovered by which platinum scraps could be melted by the electric current, and yet retain the qualities of pure platinum?

Dr. Custer stated that when platinum was fused on a block of carbon, so much carbon was taken up, the resulting metal was changed similarly to the conversion of iron into steel, having the qualities of platino-iridium. To retain the softness and ductility of the platinum it should be fused in lime, when it will equal the new platinum as when first bought.

Dr. Tileston inquired how Dr. Custer's arc-light illumination of the electric oven compares with the method employed for illuminating the Downie furnace?

Dr. Custer replied that it differs as much as the arc light differs from an incandescent light.

Dr. Molyneaux spoke of the importance of being able to recognize with exactness the fusing point of porcelain. With more accurate methods and less guesswork, more porcelain work will be done and better results will be attained.

Section II. The report of this section was devoted to a brief review of the new text-books, of which the past year has been so prolific. The committee suggest that in future editions of these works and in new text-books a glossary of terms employed be inserted, as a feature calculated to be of great value to the student; one of the great needs of to-day being a comprehensive dictionary of dental terms, giving the proper orthography, punctuation, etymology, and definition.

The section has decided to take the first steps in this direction, and asks the co-operation of the entire National Association, suggesting that the chairman and secretary of each of the ten sections take steps to secure a list of all the important words in their respective branches, sending the same to Section II. at as early a date as possible.

Section II. offered a short paper, by Dr. William Ernest Walker, of Pass Christian, Miss., entitled "Cast and Model," of which a brief abstract follows:

CAST AND MODEL.

Dr. Walker spoke of the confusion which has existed in the past with regard to the use of the terms *cast* and *model* as used in prosthetic dentistry, which led to the recommendation, by the Committee on Nomenclature of the American Dental Association, that the use of the term *cast* be discontinued, and that the term *model* be used exclusively.

Dr. Walker had found, however, a very positive necessity for the retention of the two terms, in order to avoid descriptive detail in dealing with his students and laboratory assistants. He therefore makes a distinction, as follows: The first step is the *impression;* this gives a matrix, in which is poured the *cast,*—which is not a model of anything, but which, *as a cast,* is ready for use in the construction of a vulcanite, celluloid, or cast-denture. If, however, swaged work is intended, the cast is, by the proper manipulation, *converted into* a *model* by so shaping it that it will represent the metal die that is to follow.

The successive steps, then, are, first, "impression;" second, "cast;" third, "model;" fourth, "sand-matrix;" fifth, "die;" sixth, "counter-die." This consecutive series is readily fixed in the mind of the student; each step, as named, representing a definite stage in the production of an artificial denture; a well-defined object, to which neither of the other terms in the series can be applied. If the student or laboratory man is told to make a "cast," he simply pours the plaster into the impression; but if told to make a "model," he first pours a cast, and then trims it into such shape that it becomes a model for the "die" which is to follow.

DISCUSSION.

In the discussion of the paper, the author was sustained in his position by Drs. Goddard and Barrett.

Dr. Goddard said he was very glad to hear the paper read, as it covers clearly the points that he makes in his lectures on prosthetic dentistry, and he moved that the Association approve the distinction made in the paper.

Dr. Guilford took exception to this, saying that he had gone over the ground very carefully. The Committee on Nomenclature had thought it wise to use one term, in place of two, whenever it was possible. The only difference between a cast and a model, in prosthetic dentistry, is that of a half-inch in height, which is not enough to necessitate the use of an additional term. It is better to call both "models," even though, strictly speaking, one is not always a model which is to be copied. In teaching prosthetic dentistry for many years, he had never found any confusion to arise from this method of designation.

Dr. Molyneaux said that for years he had made substantially the same distinction, and had argued in favor of that distinction at the Asbury Park meeting, but had yielded to the recommendation of the committee.

Dr. Goddard contended that more difference existed between a cast and a model than that pointed out by Dr. Guilford; the cast is not only increased in height, but it must be trimmed, in order that it may drop readily from the moulding-sand when it is to be used as a model.

*Dr. Barrett.*—Cast is the proper noun to be used to designate the object of the process of *casting;* we do not *cast* a model. A model must be manipulated with the fingers; a cast is the result of mechanical crystallization. We are working for the world, and a final decision on such points should not be reached by snap-judgment. We are not prepared to vote intelligently until we have given the subject more thought.

Dr. Molyneaux said that his argument on the point in question had been carefully prepared for presentation at the Asbury Park meeting, but it had not been taken up; he thought it should have received more consideration.

Dr. Barrett moved, as an amendment to Dr. Goddard's motion, that the paper be referred to the Publication Committee, and that the point in nomenclature be referred back to Section II. for further consideration.

Dr. Goddard withdrew his motion in favor of that of Dr. Barrett.

After some further discussion, Dr. Barrett withdrew his motion, which was, however, renewed by Dr. Weeks in substantially the same form, and adopted.

Section III. reported better organization of sectional work, and recommended the encouragement of the development of this section, as it has, perhaps, the largest element to draw from in the Association.

The section presented one paper, by Dr. B. Holly Smith, a brief abstract of which is presented on page 702.

Section IV. offered no report, neither chairman nor secretary being present.

Section V., Dr. Cassidy, chairman. Dr. Cassidy read a report on "Dental Materia Medica," of which we give an abstract, as follows:

The deluge of drugs of antiseptic quality, following the acceptance of the bacterial genesis of many oral diseases, has culminated in a tendency to ignore the very new remedies, and investigate more thoroughly the virtues of those more or less well known, which are comparatively simple in their chemical and therapeutic action, time-tried and reliable, non-secret, and not even proprietary.

The professedly new introduction, formaldehyde, is the active principle of the earth of ant-hills, which, from time immemorial, has been gathered by the people of continental Europe, and used successfully, by local application, in the domestic treatment of swellings, bruises, suppurating sores, abscesses, rheumatic pains, etc.

Dr. Cassidy stated that he introduced this compound to the American Dental Association in 1894. It was then classed as a combined disinfectant, antiseptic, and germicide. These claims have been found to be based on solid truth, but its irritant properties render it unpleasant to use in the mouth without a corrective. Some cases of sloughing of the gums from its use, even when diluted, are reported.

It is, however, a very complete sterilizer for extracted teeth intended for use in technic instruction, and there is no agent known which will so thoroughly and safely sterilize burs, excavators, etc., dipping them, after cleansing, in full strength formalin, and wiping dry.

The latest phase in cataphoresis is the application of the definite rules of electrolysis, so that the proper pole to be used in connection with the different cataphorics may be readily selected. Non-conductors also, as alcohol, chloroform, phenol, tincture of iodine, etc., can be used successfully, the pressure of the current itself, as conducted by the tissues from pole to pole, inducing these agents to penetrate farther than by mere absorption.

Dr. Cassidy concluded his report with a criticism of an article published in the June issue of the *Dental Cosmos* (from the *Journal of the British Dental Association*), detailing a series of experiments by Drs. Kemp and Brush, on nitrous oxide, proving its specific anæsthetic influence as distinct from any inert gas. Dr. Kemp says, "With regard to the general metabolism, the result of these researches is to show positively that carbon dioxide in the arterial blood is greatly diminished, and is *not* increased, so that any theory involving the dependence of anæsthesia on a retention of carbon dioxide within the system is in opposition to the direct findings of these experiments." Dr. Cassidy criticises the implied statement that carbon dioxide escapes from the body by means of the arteries. He suggests that if Dr. Kemp examine on the venous side of the capillaries instead of on the arterial side, after giving his dog an average dose of nitrous oxide he will find strong evidence of the accumulated carbon dioxide. Nitrous oxide, when inhaled, goes through the arterial circulation unchanged, superseding, for the time being, iron,—the oxygen carrier of the body; when the capillaries are reached, it gives up its oxygen to form the same combination with carbon, hydrogen, etc., that atmospheric oxygen produces, but to a greater extent:

The carbon dioxide developed thus cannot escape freely, for the reason that the reduction of the ferric oxide in the arterial blood not being accomplished, the iron is unprepared to convey the carbon dioxide as ferrous carbonate to the lungs. If, however, either free oxygen or air be administered simultaneously, or at short intervals, one of the physiologic functions of iron is restored sufficiently to prevent cyanosis, and the animal may thus be kept indefinitely in the anæsthetic condition, without danger of asphyxiation.

It is gratifying to know that this—the carbonic acid theory of anæsthesia by nitrous oxide—is being investigated by such an institution as the Johns Hopkins University.

Passed without discussion.

Section VI. not ready to report.

Section VII. Anatomy, Pathology, and Surgery. W. C. Barrett, chairman.

Dr. Barrett, in his report, referred to the collection of specimens in comparative odontology, exhibited last year at Old Point Comfort, as being probably the finest ever gotten together.

He reviewed briefly the literature of the past year on the subjects germane to the section.

Of the papers and lantern exhibitions by Dr. J. L. Williams, of London, and M. H. Cryer, of Philadelphia, the first apparently proves that the vascular supply of the tooth-pulp must come from the pericementum, and that the old cuts of the text-books, representing the inferior dental artery as giving off branches which enter at the apical foramen of each tooth-root, are misrepresentations of the real condition, the pericemental membrane being, in fact, the placental membrane, which supplies the tooth-pulp with nutriment. The preparations of Dr. Cryer, on the other hand, would seem to prove that the old text-books were correct; but neither in the exhibition of Dr. Williams nor in that of Dr. Cryer was anything conclusively presented. Dr. Williams must prove that his preparations positively bear the interpretation placed upon them by him; Dr. Cryer must determine, by injections, that there is direct communication of the dental pulp with the dental artery before his deductions can be accepted as final.

In dental surgery nothing specifically new has been presented. Dr. Brophy's operation for the radical cure of cleft palate seems to continue successful. The most surprising fact noted in connection with this operation is that, although bringing together the divided palatal walls necessarily contracts the upper jaw from one-third to one-half, which would be expected to result in a permanently deformed upper jaw, with the superior teeth inside of the lower ones, yet this has not been the result in a single case. The superior jaw has developed into the normal size, the deciduous teeth erupting in perfect occlusion,—a mysterious process in nature, and a lesson in surgical procedure that may well engage earnest attention.

In pathology nothing has been definitely settled in regard to pyorrhœa alveolaris. The advocates of the different hypotheses still hold to their pronounced views, but have not demonstrated their claims to the satisfaction of others.

Difference of opinion still exists as to the use of drainage-tubes in the treatment of antral troubles. The arguments for and against are reviewed in the report, and the conclusion reached that a cavity that must be drained should be left permanently open so long as there is any pus-formation; that tubes, plugs, and tents are necessarily mischievous in their nature, as tending to retain the discharge and perpetuate the septic condition; the presence of a tube also causing exuberant granulations around its borders.

The precise nature of the action of arsenic upon vascular tissue and the changes succeeding its application to the dental pulp have never been sufficiently demonstrated nor explained. It has been claimed that the death of the pulp is caused by congestion, producing strangulation at the foraminal apex; but this cannot be the case in a partially developed tooth, to which arsenic proves as fatal as in the case of the completely formed root. Its application to the mucous membrane of the buccal cavity produces equally serious results. The action of arsenic on the dental pulp is not essentially different from its effect on internal tissue when taken internally, and seems to indicate special dynamic energy. The minimum amount required for pulp-devitalization has not been definitely ascertained, but the mistake usually made is that of using an unnecessarily large amount, causing an intensely inflamed, irritated condition of the corpuscles of the cementum at the extremity of the root, creating an exceedingly sensitive point, apparently non-devitalized pulp tissue at the apex, to which a second application is usually made. This simply serves to destroy the vitality of the apical cementum, often inducing serious trouble. If the tissue in the pulp-chamber is found devitalized, there is no question about that in the root-canals. If a sensitive point is reached, instead of making a second application of arsenic, the arsenic present should be neutralized by an application of dialyzed iron, and anodynes employed until all further danger from the poison is averted. The root canals can then be cleansed and filled.

The new books on subjects germane to Section VII. were considered by Section II.

DISCUSSION.

Dr. Brophy said, in regard to the treatment of diseased antrum, the opening must naturally be made at the most dependent part,—the canine fossa. In serious cases it must be sufficiently large to permit of thorough exploration and ocular observation. A canula, with a flange on the outside to hold it in place, is essential to thorough irrigation of the cavities. A small opening is of no avail, except for the temporary evacuation of the cavity.

Dr. G. V. I. Brown prefers to pack the cavity with antiseptic gauze, the tube being a source of disease. The gauze packing does not interfere with the process of healthy granulation, and has not the disadvantages of the drainage-tube.

Dr. E. H. Smith said that if the disease were due to dental irritation, proper treatment of the dead tooth, thus removing the cause of the disease, was often sufficient, evacuating the antrum through the root-canal of the tooth, without making a large opening.

Dr. C. N. Peirce agreed with Dr. Smith.

Dr. Crawford said there was no field in which more mistakes in diagnosis and treatment are made than in antral trouble. He has seen no less than five cases, starting from a tooth and opening into the nasal cavity, which had been diagnosed and treated as antral trouble.

Dr. Brophy said that in serious chronic cases a large opening is necessary, but in a large majority of cases the repeated washings and drenchings do more harm than good.

PYORRHŒA ALVEOLARIS.

Dr. Patterson said that he did not believe there was such a thing as pyorrhœa alveolaris without deposits. A *shaving process* will often remove deposits which the probe fails to find, the cure following showing that these minute deposits were the cause of the trouble. Small patches, not to be seen except with a strong eye-glass, are as irritating to the pericemental tissue as larger accumulations.

Dr. Barrett said that where there are pockets there are always deposits; but in many cases, where there is a wasting, purulent condition of the pericemental membrane, there may be no deposits.

THE BROPHY OPERATION FOR CLEFT PALATE.

Dr. Patterson said that he had formerly been pronounced in his opposition to the Brophy operation for cleft palate. He wished now to go on record as in favor of it, having been convinced by a knowledge of three successful cases. He had never seen, by other methods, anything that approached a success, the only object of such operations being the improvement of the articulation, which by the Brophy operation is a success.

Dr. Barrett considers the operation the grandest triumph of our profession. As the Great Physician anointed the eyes of the blind and they saw, so modern oral surgery touches the lips of the dumb and they speak. For the first time in the history of surgery, *cleft palate can be cured.*

Dr. Brophy was invited to prepare an address on the subject of surgical operation for cleft palate for the next meeting, and promised, provided the meeting be held within a convenient radius of Chicago, to present several small children who had been operated upon at a very early age, that the results of the operations may be seen.

Section VII. offered three papers,—(1) "Anatomy: A Critique," by William Ernest Walker; (2) "The Comparative Method of teaching Dental Anatomy," by A. H. Thompson; (3) "Paper illustrated with Slides," by M. H. Cryer,—which were next read.

Dr. Walker directed attention to a few errors which have crept into the 1897 edition of Gray's "Anatomy," to which the attention of students should be directed.

In the definitions of the names of the surfaces of the teeth (page 932), they are given as labial, lingual, buccal, distal, and proximal. Distal is defined as "the surface away from the mesial line," and proximal as being the reverse of distal. Dr. G. V. Black's definition of proximate or proximal, "applied to a surface of a tooth (either distal or mesial) which is next to another tooth," is more satisfactory. Mesial, however, is not given as the name of a tooth surface, neither is it anywhere defined, although in the text it is universally substituted for proximal, as defined. (See page 933: "The mesial and distal surfaces are triangular," "short mesial" and "long distal" cutting edges, etc.)

Looking up the word mesial in the dictionaries, the student would be led to believe that the mesial plane or line must pass antero-posteriorly between the superior central incisors parallel with the tongue, and that consequently the surface of the tooth which is towards the mesial line must face the sides of the tongue.

This was certainly not the idea intended to be conveyed by the definition of the proximal surface of a tooth.

Dr. Black's definitions of distal and mesial, as accepted by the Committee on Nomenclature of the American Dental Association (*Transactions*, 1895) are more perspicuous: "Distal: away from the median line of the face, following the course of the dental arch;" "Mesial: towards the median line of the face, following the curve of the dental arch."

On page 935 we read, "The movement of the human mandible is forward and downward, the resultant of these directions being an oblique line, upon an average of 35 degrees from the horizontal plane." As authority for this statement, a foot-note refers to "W. E. Walker, *Dental Cosmos*, 1896." The statement, as given, is misleading, and is not a correct deduction from the article referred to. *Mandible* should read "mandibular condyle;" "35 degrees from the horizontal plane" should read "40 degrees from the facial line." A plane perpendicular to the facial line could be termed *horizontal* only while the latter was absolutely vertical. The plane of condyle movement forms an average angle of 35 degrees with the plane of occlusion, the latter forming an average angle of fifteen degrees to a plane perpendicular to the facial line, which plane forms an angle of from 40 to 50 degrees with the plane of condyle movement.

Dr. Walker next takes issue with the statement that when the lower jaw is advanced until the cutting edges of the incisors are in contact, the molars also come in contact, thus relieving the strain upon the incisors. This statement, first noted in Bödecker's "Anatomy and Pathology of the Teeth," and repeated in more definite terms in the 1897 edition of Gray's "Anatomy," is amplified in Dr. Burchard's recently issued "Anatomy and Pathology of the Teeth," as follows: "While the jaws are in position, with the incisors in occlusal contact, they do not bear alone the stress of whatever muscular force is applied; but it will be seen that the distal cusps of the third molars, the highest points of the dental arch, advance and meet the distal cusps of the upper second molars, so that when the incisors are in edge-to-edge occlusion, although all of the other teeth are separated to an extent governed by the overbite, the dental arch is supported posteriorly by contact of the last molars, thus preventing undue stress upon the incisors."

Dr. Walker said that his studies of articulation and occlusion had convinced him that this statement is not based on fact. In order to test the point, he had on one occasion taken casts of students' mouths at the Dental College, taking them as they came, rejecting only those mouths in which very many teeth had been extracted, until he had taken and was prepared to exhibit thirty-three casts of jaws with the teeth in edge-to-edge occlusion. Of these, seven were excluded because of abnormality, there being no overbite in the position of rest. Of the twenty-six left, twenty-two had the molars *entirely free* when the incisors were thus placed end to end. One has contact of the molars on the right side only, where the second and third molars have come forward, due to the loss of the first molar. The second case, in which there is molar contact, has lost the left upper second bicuspid and left lower first molar; also the right lower second bicuspid and the first molar. The third of the "contact" cases has no bicuspids and only one molar on the left lower side, and shows great weight of the incisors. He has also casts of three unusually symmetrical mouths, with the teeth both in normal occlusion, and also in the *biting position.* These casts also fully sustain the position that, as a rule, the molars are not in contact when the incisors are in the edge-to-edge position. He has concluded that the idea must have originated in a misunderstanding by Dr. Bödecker of Dr. Bonwill's advice to construct *artificial dentures* with what he calls "a balancing articulation," making the artificial molars meet at the same time that the incisors meet, in order to support the plate and prevent its displacement in mastication.

The error which appears to have thus originated with Dr. Bödecker has apparently been perpetuated by subsequent writers, without any effort being made to either confirm or refute it, as far as known to the writer, who requests that, if investigations have been made on this point, the results be made known, that a definite conclusion may be reached as to the correctness or otherwise of the statement with which he takes issue.

(To be continued.)

